# Proteomic Analysis of *Bacillus thuringiensis* Strain 4.0718 at Different Growth Phases

**DOI:** 10.1100/2012/798739

**Published:** 2012-04-29

**Authors:** Xiaohui Li, Xuezhi Ding, Liqiu Xia, Yunjun Sun, Can Yuan, Jia Yin

**Affiliations:** Key Laboratory of Microbial Molecular Biology of Hunan Province, College of Life Science, Hunan Normal University, Changsha 410081, China

## Abstract

The growth process of *Bacillus thuringiensis* Bt4.0718 strain was studied using proteomic technologies. The proteins of Bt whole cells at three phases—middle vegetative, early sporulation, and late sporulation—were extracted with lysis buffer, followed with separation by 2-DE and identified by MALDI-TOF/TOF MS. Bioactive factors such as insecticidal crystal proteins (ICPs) including Cry1Ac(3), Cry2Aa, and BTRX28, immune inhibitor (InhA), and InhA precursor were identified. InhA started to express at the middle vegetative phase, suggesting its contribution to the survival of Bt in the host body. At the early sporulation phase, ICPs started their expression. CotJC, OppA, ORF1, and SpoIVA related to the formation of crystals and spores were identified, the expression characteristics of which ensured the stable formation of crystals and spores. This study provides an important foundation for further exploration of the stable expression of ICPs, the smooth formation of crystals, and the construction of recombinant strains.

## 1. Introduction

*Bacillus thuringiensis *(Bt) is a ubiquitous Gram-positive, spore-forming bacterium that produces parasporal crystalline inclusions named insecticidal crystal proteins (ICPs) during its sporulation phase [1, 2]. Its insecticidal activity is largely attributed to its insecticidal crystal proteins, which were used in the development of important microbial insecticidal pesticides [3] widely used in agriculture and forestry [4].

 Genome sequencing and annotation of *Bt. spp. kurstaki *97-27 was achieved [5]. Compared to genome studies, proteome investigation can provide an insight into factors such as protein presence, abundance, and posttranslational modification. With such information, we can gain an understanding of its physiology, pathogenesis, and mechanism of avoiding host immune systems. Two-dimensional gel electrophoresis (2-DE) combining with mass spectrometry (MS) has been used to study many bacteria, such as *E. coli *[6–8], *Bacillus anthracis *[9, 10], and *Bacillus cereus *[11, 12].

 There have been several studies on *Bacillus thuringiensis *at the protein level [13, 14]. However, most of these efforts have been centered on the identification of ICPs [15] and bacteriocin [16, 17] using SDS-PAGE, Western blot, and MS. Meanwhile, 2-DE was used to identify Bt crystal proteins [18].

To date, characterization of the process of spore and crystal formation of *Bacillus thuringiensis *has not been reported. The aim of our study was to identify the proteins expressed during the growth of Bt cells and to find more bioactive components including insecticidal crystal proteins (ICPs) in the Bt4.0718 strain. In this study, several kinds of bioactive factors such as Cry1Ac(3), Cry2Aa, InhA, InhA precursor, and bacillolysin were identified in Bt4.0718 strain. The expression differences of the bioactive factors between the three phases and their effect on virulence of bacteria cell were analyzed. Threonine synthase, CodY protein, ORF1, CotJC, OppA, and SpoIVA which may take part in or regulate the expression of crystal proteins or spore formation were identified, some of these proteins have not been reported by previous studies on Bt, and their sequences and functions in *Bacillus thuringiensis* need to be studied in depth. This research provides an important foundation for the steady expression of crystal proteins in Bt and the construction of recombinant Bt strains with high toxicity.

## 2. Materials and Methods

### 2.1. Culture and Observation of Bt Cells

*Bacillus thuringiensis* 4.0718 strain was used in this study. It produces two major kinds of parasporal crystals, Cry1 (130 kDa) and Cry2 (65 kDa) [19]. Cells were grown in fermentation medium at 30°C with rotary shaking at 200 rpm. Bt cells were collected every two hours for 28 hours after cells had been transferred into the fermentation medium. The cell growth density was determined at OD600 nm by using blank culture medium as the control. The changes in the Bt cells during their life cycle were monitored by observation of samples under contrast-phase microscopy.

### 2.2. Preparation of Protein Samples

Bt Cells were harvested at different growth phases (middle vegetative phases (T_1_), early sporulation phase (T_2_), and late sporulation phase (T_3_) and centrifuged for 9 min at 9,600 g at 4°C. The pellets were washed four times for 7 min at 9,600 g with low-salt washing buffer. The pellets were vacuum-dried and stored at −20°C. Dried pellets weighing 0.036 g were resuspended in 1 mL Milli-Q water containing 1 mM PMSF and ruptured by sonication for 10 min at 0°C. After adding 15 U RQ1 DNase and 15 *μ*L 10 mM RNase A to 540 *μ*L of the lysed cell suspension, which was then kept in ice-cold water for 1 h, solid thiourea, CHAPS, urea, Tris and DTT were added to final concentration of 2 M, 4%, 8 M, 40 mM, and 60 mM, respectively. The solution was kept at 4°C for 30 min and then at room temperature for 30 min to solubilize proteins efficiently. It was then centrifuged for 15 min at 16,000 g to precipitate the insoluble components. The supernatant was collected, and its protein concentration was determined using the Bradford method [20] and the 2D Quant Kit (GE Biosciences) [21] and then stored in aliquots at −70°C till used for 2-DE.

### 2.3. 2-DE

2-DE was performed according to the methodology described previously [22]. Isoelectric focusing was performed in Immobiline IPG strips (18 cm) on an IPGphor system (GE, formerly Amersham Pharmacia Biotech). Protein samples of 1 mg were mixed with a rehydration solution containing 8 M urea, 2 M Thiourea, 4% CHAPS, 40 mM Tris, IPG buffer (0.5% V/V, pH 4–7), 18 mM DTT, and traces of bromophenol blue, to a total volume of 350 *μ*L. The mixture was pipetted into the strip holder. Isoelectric focusing (IEF) was carried out at 20°C on the IPGphor unit under the following steps: (1) hydration for 12 h, (2) 200 V for 1 h, (3) 500 V for 1 h, (4) 1000 V for 1 h, (5) Grad 8000 V for 0.5 h, and (6) 8000 V for 7 h. After focusing, the strips were soaked for 15 min in reduction solution (2% SDS, 6 M urea, 30% glycerol, and 125 mM DTT) followed by 15 min in alkylation solution (2% SDS, 6 M urea, 30% glycerol, and 125 mM iodoacetamide). The SDS-PAGE step was performed in 10% polyacrylamide gel run on a Protean II system (Bio-Rad, Hercules, CA, USA) at 10°C. After 2-DE, gels were stained with a Coomassie Brilliant Blue (CBB) solution. Spot detection, quantification, and matchset were performed using Melanie 6.0 software.

### 2.4. In-Gel Protein Digestion

The CBB-stained protein spots were cut out from the gels and washed twice with 50 *μ*L water and then destained three times with 50 *μ*L 25 mM ammonium bicarbonate and 50% ACN for 30 min at 37°C. The destained gel pieces were washed with 25 mM ammonium bicarbonate, 50% ACN, and 100% ACN in turn. These dehydrated pieces were lyophilized in a vacuum centrifuge. Vacuum-dried Gel pieces were reswolled with 5 *μ*g 25 mM ammonium bicarbonate containing trypsin at low temperature for 45 min followed by added buffer. After incubation at 37°C for 16 hours, the supernatant was transferred into another tube and 50 *μ*L 67% acetonitrile (ACN) was added to extract peptides in the gel pieces. After 3 h incubation at 37°C, extracted supernatant was combined with the first one. The combined supernatant was lyophilized for MS.

### 2.5. MALDI-TOF/TOF MS and Protein Identification

The digested peptides were solubilized in deionized water containing 0.1% TFA. A 1 *μ*L sample was mixed with 0.5 *μ*L matrix solution (saturated solution of CCA in 50% ACN and 0.1% TFA). The mixture was applied to a target well and introduced into the mass spectrometer after air-drying. Bovine serum albumin products were used for external calibration.

For proteomic identification, MS/MS spectra were searched by MASCOT (Matrix Science, http://www.matrixscience.com/) using the no-redundant NCBI Gram-positive bacterial sequence database. Mass tolerance was set at 50 ppm for the mass of peptide precursors and at 0.6 Da for the mass of fragment ions. Carbamidomethylation of cysteines and methionine oxidation were set as the fixed and variable modifications, respectively.

## 3. Results

### 3.1. Morphology of Bt Cells, Spores, and Crystals at Different Growth Phases

**Figure 1:**
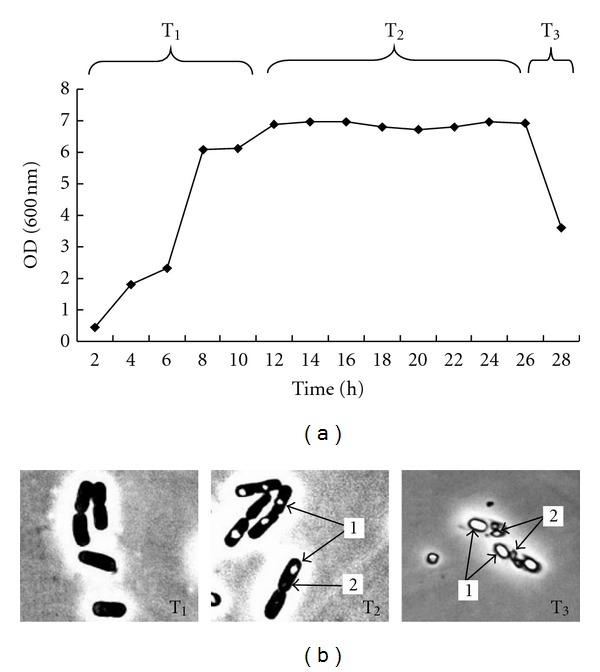
(a) Growth curve of *Bacillus thuringiensis* strain 4.0718. T_1_, middle vegetative phases T_2_, early sporulation phase T_3_, and late sporulation phase. (b) Morphology of* Bacillus thuringiensis* cells at different phases observed through phase contrast microscopy. 1: spores; 2: crystals.

The growth curve of Bt4.0718 according to the optical density determinations at OD600 nm is presented in Figure 1(a). The optical density increased exponentially from 2 to 12 h (T_1_), indicating that bacteria during this period are in the logarithmic phase. From 12 to 26 h (T_2_), the optical density changes little, showing that bacteria during this period are in the stationary phase. After 26 h (T_3_), the optical density value declined sharply, which means that bacteria reached the death phase.

These observations (Figure 1(b)) showed that, at T_1_ phase, no spore or crystal could be observed. At T_2_ phase, spores of atactic shape existed in Bt cells while the crystals were absent in the majority of cells. At T_3_ phase, cell walls became thin; the mature spores and crystals with orderly shape were present in the cells, making up most of the cell volume.

### 3.2. Theoretical Protein Map

**Figure 2:**
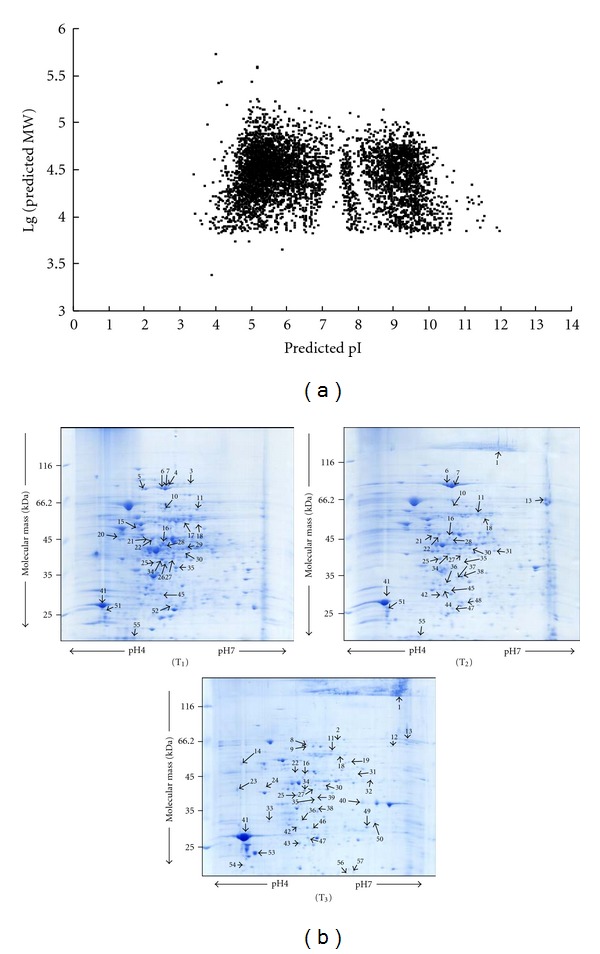
(a) Representation of 2-DE gel separation of the total proteins of whole cell of *Bacillus thuringiensis* according to predicted pI and molecular masses. (b) 2-DE maps of the proteins of whole cells at T_1_, T_2_, and T_3_ from Bt4.0718 strain. The protein spots marked with arrows were identified by MALDI-TOF/TOF MS.

The theoretical 2-DE maps of pH 0–14 were constructed according to the protein information on the SWISS-PROT website to show the distribution of the global predicted proteins on the gel. To simulate protein mobility during 2-DE, the *y*-axis was drawn on a logarithmic scale to represent the migration trend during SDS-PAGE, and the *x*-axis was drawn on a linear scale to imitate protein mobility during IEF (Figure 2(a)). As shown in the calculated gel, more than 60% of the proteins of the Bt were found to have a theoretical pI between 4 and 7, similar to other bacterial species [9, 23]. According to the theoretical distribution and due to the difficulty in handling the alkaline protein, the pH range of 4–7 was chosen as the standard analytical window in this experiment.

### 3.3. 2-DE Analysis and Protein Identification

In order to identify more bioactive factors and proteins that may be involved in the regulation of crystal and spore formation, 2-DE was used to compare the proteomic profiles of proteins of Bt4.0718 strain at different growth phases. The protein profiles of the 2-DE gels are shown in Figure 2(b). After image analysis and spot quantification by Melanie 6.0 software, a total of 346, 299, and 343 protein spots were detected on the 2-DE gels of the three phases of T_1_, T_2_, and T_3_, respectively. The matching ratios of 2-DE gels of protein samples of T_1_ and T_3_, to 2-DE gel of the protein sample of T_2_, which was set as the reference gel, were 70% and 60%, respectively. All the protein spots with different expression were excised but only 56 of them presenting 49 proteins were identified using MALDI-TOF/TOF MS and Gram-positive bacteria database search. Meanwhile, an important protein without significant regulation between the three different phases was identified. There are same proteins (Table 1) identified in the three phases. According to the functions of these identified proteins (Table 2), all the protein spots were categorized into five major groups: bioactive factors, metabolism, crystal and spore formation, amino acid and protein synthesis, and other functions.

## 4. Discussion

### 4.1. Physiology of Bt4.0718

One goal of proteomic studies consists in providing a global comprehensive view of cellular physiology and cellular adaptation reactions [23]. In the proteomic studies of *Bacillus*, the proteins of cells at mid-exponential phase had been separated and identified to resolve the rapid growth of the entire spectrum of proteins [9, 24]. Also, proteins extracted from cells growing in cultures with high salt concentration [25] or facing other high-pressure environments [26] were compared with proteins from cells in normal cultures using the proteomic approach, to find proteins closely related to resistance to environmental stress. The function of these proteins was analyzed to understand the internal regulation reaction. In our study, proteins extracted from cells at different growth phases were separated and identified. The proteins identified from cells at T_1_ were mainly enzymes involved in metabolism, similar to the protein profiling of *Bacillus subtilis* [24]. To study the proteome of whole cells in Bt was to study the changes of protein expressions of Bt cells during their lives. And the formation and maturation of spores and crystals are the direct results of protein expression changes. Both of them are important parameters according to appropriate phases we chose as research targets.

The regulation reactions were also studied by analyzing proteins with different expression during the lifetime of Bt4.0718. The results showed that, at T_1_, the enzymes involved in metabolism sustained rapid cell growth, and crystal and spore formation did not commence. At T_2_, some proteins participating in the citric acid cycle enzymes were downregulated; spores were formed while ICPs started to express in the phase, which were regulated by the expression of threonine synthase upregulated significantly, and CodY inhibited the expression of proteins at the early stability phase, providing a large number of raw materials for ICPs. Therefore, although at T_2_ the nutrition for growth of Bt cells has already begun to be restricted, ICPs can have a fast and efficient expression. As the components of nutrients in the medium changed, organic tolerance proteins started to express, helping cells to adapt to the environment, and nutrition is also the important prerequisite of spore formation. At T_3_, the mature spores and crystals were formed, providing a lot of pressure on space in the cells. The study found that PspA protein was induced to express, which played an important role in the survival of adversity [27], and organic tolerance protein has a certain expression in Bt cells. The expression could help Bt cells adapt to the environment. This is the first preliminary study of the Bt cell physiology by use of proteomic technologies, and the intracellular physiological changes during the lifetime of Bt were analyzed, laying an important foundation for the in-depth study of Bt physiology.

### 4.2. Bioactive Factors and Bt Virulence

Besides the analysis of Bt cell physiological change during its lifetime, many bioactive factors such as insecticidal crystal proteins (ICPs), immune inhibitor A (InhA), and InhA precursor separated by 2-DE in the cells of Bt4.0718 were analyzed, which will help to understand the relationship between their expression and Bt virulence. 

In Bt4.0718, ICPs included Cry1Ac(3), BTRX28, and Cry2Aa which were separated and identified by SDS-PAGE (figure not shown). The clustalX analyses showed that BTRX28 shared 98% homology with Cry1Aa. At T_2_, ICPs started to express, and the protein concentration was the highest or maximal. Therefore, ICPs were the most important part of Bt virulence at the sporulation phase. The proteins classified into Cry1 can specifically kill insects of Coleoptera, while Cry2 can specifically kill insects of Diptera. The kinds of ICPs in Bt4.0718 strain determined its insecticidal spectrum, consistent to the results of bioactivity tests [19]. InhA is a kind of secret neutral metalloproteinase containing zinc metal [28], which can destroy antibacterial peptides secreted by insect hosts, such as cecropins and attacins from the immune hemolymph of *Hyalophora cecropia *[29]. Consequently, it can impress the humoral immune system of insect hosts. Because of this characteristic, InhA can protect Bt cells by prohibiting the lysis action of antibacterial proteins to Bt cells [30]. In our study, at T_1_ InhA and InhA precursor protein started to express when the ICPs did not express, at T_2_, the expression amount of InhA and InhA precursor protein was the greatest, but at T_3_, they could not be identified from 2-DE gel. The investigation suggested that, after invading the insect host, Bt expressed InhA that inhibited the cell lysis action of antibacterial peptides produced by the host, in favor of the subsistence of Bt in the host body. It laid a basis for the formation of crystals and spores and the exertion of Bt virulence at the sporulation phase. Camelysin and bacillolysin are also important bioactive factors. Camelysin first discovered in *Bacillus cereus *(Bs) was about the harm of *Bacillus cereus* to humans [31]. In *Bacillus thuringiensis *subsp.* israelensis*, the sequence of a putative protease shared high homology with camelysin of the closely related Bs species. Recently it is reported [32] that the camelysin had a positive effect on regulating the expression of InhA. The protease can make cleavage between amino acid residues 34 and 35 of Cyt2Ba, to obtain rather pure and active toxin species quickly and simply [33]. In this study, three camelysins were identified in Bt4.0718; the amount of one camelysin remains unchanged while the other two only synthesized at T_3_. It demonstrated to some degree that camelysin in Bt is not only related to spore formation but also to the virulence activity of crystals. Bacillolysin can catalyze the hydrolysis of peptide bonds of amino ground terminals of leucine or phenylalanine. Some studies have shown that thermolysin-like metalloproteinases such as aurelysin, pseudelysin, and bacillolysin presented virulence factors of diverse bacterial pathogens. These particular microbial metalloproteinases mediate sensing of invading microbes and elicit innate immune responses in insects. They played a predominant role as virulence factors and promoted the development of bacteria or fungi within the infected hosts [34]. The functions of bacillolysin in Bt should be studied in depth. It was found that at any phase, the expression of ICPs was accompanied with other bioactive factors' expression, though Bt virulence was sustained mainly by ICPs. It was demonstrated that these bioactive factors with weak biological activity played an important role in Bt virulence.

 The diversity of bioactive factors of Bt4.0718 provided a scientific basis and practical ideas to study its insecticide mechanisms to broaden its insecticide spectrum and to enhance its killing effect.

### 4.3. Regulation Reaction in the Formation of Crystals and Spores

The important role of regulator was usually studied with mutation methods. For example, the important role of SpoIVF involved in the crystal formation process of Bt was researched by the observation of crystal and spore formation in a SpoIVF deficient mutant [35]. Also, proteomic technologies were used to detect the protein expression differences between mutant and wild strains [14]. In our study, parts of regulators associated with the formation of crystals and spores, such as OppA, CotJC protein, SpoIV, and ORF1, were separated and identified by proteomic technologies. The expression and function of the regulators were analyzed to gain a relatively clear understanding about their roles in the formation of crystals and spores during Bt's lifetime. As crystals and spores began to form at T_2_, certain proteins associated with crystal and spore formation were identified at T_2_ first. CotJC protein and OppA were identified at T_2_. In *Bacillus subtilis *(Bs), CotJC protein manipulation is necessary for the normal formation of spore coat, and some of them are even the composition of spores [36, 37]. In our study, CotJC protein in Bt was only expressed in the process of spore formation, which noted that the role of CotJC protein in Bt should be similar to that in Bs, but it needed further confirmation. Oligopeptide transport system (Opp system) in Bs is important at the early sporulation phase [38]. It was found that in *Bacillus thuringiensis* with *opp* mutation, the rate of sprouting was reduced, and the phosphorylation of Spo0A can be controlled by Opp protein in both Bt and Bs [39]. The oligopeptide ABC transporter protein and oppA identified in this study both belong to the oligopeptide transporter system. The two proteins were specifically expressed in the sporulation phase, so that they may play an important role in the formation of spores in Bt4.0718. At T_3_, uncharacterized 20 kDa protein in CryB1 5′ region (ORF1) and phase IV sporulation protein A (SpoIVA) started to express specifically. The gene of ORF1 (*orf1*) is one of open reading frames at upstream of *cry2A* operon; *orf1* is 33% homology with the *p19* gene of cry11Aa operon, but the expression of *orf1* could not help the expression of Cry2Aa. However, Ge proved that the participation of ORF2, the gene of which (*orf2*) is also another open reading frame next to *orf1 *at upstream of *cry2A* operon, cannot only enhance the expression of Cry2Aa but also help the formation of crystals [40]. ORF2 duplication unit for Cry2Aa may provide attachment (matrix) or scaffold (scaffold) to the formation of crystals and can also help misfolded protein crystals refold into the right structure to immunize it to the risk of degradation, so as to enhance the protein crystal structure and stability. So it demonstrated that ORF2 ought to specifically express at T_3_ as ORF1. The results show that ICPs had started to express when spores began their formation, but the contrast-phase microscopic observation showed that when it was hard to observe the existence of crystals, the structure of spores had been clear at T_2_, but at T_3_, the structures of crystals and spores were both clearly observed. Therefore, it was suggested that the specific expression of ORF1 and ORF2 at T_3_ played an important role in the crystal formation. SpoIVA in *Bacillus subtilis *(Bs) is a conservative cytoplasmic protein which has a specialized role in the morphogenesis of spore cortex and the attachment of spore coat to the spores surface [41]. SpoIVA is expressed soon after the formation of the asymmetric septum during sporulation and acts in the mother cell compartment. Up till now, the function of SpoIVA in Bt had not been described in detail, but according to the relationship between Bt and Bs, the specific expression of SpoIVA in Bt suggested its importance during spore formation.

 Through comparative analysis of the expression differences of proteins in whole Bt cells at different growth phases, the impact on the regulation and control to the strains in the course of the formation of crystals and spores could be understood. It lays an important foundation for further exploration of the stable expression of ICPs, the smooth formation of crystal, and the application in the production in Bt recombinant strains.

## Figures and Tables

**Table 1:** The same proteins identified in the three phases.

Accession no.	Protein description
gi | 30020418	2-methylcitrate dehydratase (*Bacillus cereus* ATCC 14579)
gi | 30018379	30S ribosomal protein S10 (*Bacillus cereus *ATCC 14579)
gi | 30018375	30S ribosomal protein S12 (*Bacillus cereus *ATCC 14579)
gi | 30018386	30S ribosomal protein S3 (*Bacillus cereus* ATCC 14579)
gi | 30022730	30S ribosomal protein S4 (*Bacillus cereus* ATCC 14579)
gi | 30018397	30S ribosomal protein S5 (*Bacillus cereus* ATCC 14579)
gi | 30023498	30S ribosomal protein S6 (*Bacillus cereus* ATCC 14579)
gi | 30018489	4-hydroxyphenylpyruvate dioxygenase (*Bacillus cereus* ATCC 14579)
gi | 30018412	50S ribosomal protein L13 (*Bacillus cereus *ATCC 14579)
gi | 30018407	50S ribosomal protein L17 (*Bacillus cereus* ATCC 14579)
gi | 30018383	50S ribosomal protein L2 (*Bacillus cereus* ATCC 14579)
gi | 30022519	50S ribosomal protein L21 (*Bacillus cereus* ATCC 14579)
gi | 30018385	50S ribosomal protein L22 (*Bacillus cereus *ATCC 14579)
gi | 30260309	50S ribosomal protein L29 (*Bacillus anthracis* str. Ames)
gi | 30018380	50S ribosomal protein L3 (*Bacillus cereus* ATCC 14579)
gi | 30018392	50S ribosomal protein L5 (*Bacillus cereus* ATCC 14579)
gi | 30021657	Aldehyde dehydrogenase (*Bacillus cereus *ATCC 14579)
gi | 30021917	ATP-dependent protease ATP-binding subunit (*Bacillus cereus* ATCC 14579)
gi | 167936927	Cell envelope-bound metalloprotease (camelysin) (*Bacillus cereus *AH1134)
gi | 161511213	co-chaperonin GroES (*Bacillus cereus* ATCC 14579)
gi | 30022058	dihydrolipoamide dehydrogenase (*Bacillus cereus* ATCC 14579)
gi | 30022244	dihydrolipoamide dehydrogenase (*Bacillus cereus* ATCC 14579)
gi | 30260298	elongation factor G (*Bacillus anthracis* str. Ames)
gi | 30018377	elongation factor G (*Bacillus cereus* ATCC 14579)
gi | 30018378	elongation factor Tu (*Bacillus cereus *ATCC 14579)
gi | 30023337	F0F1 ATP synthase subunit beta (*Bacillus cereus* ATCC 14579)
gi | 67866498	InhA (*Bacillus thuringiensis serovar kurstaki*)
gi | 30022668	isocitrate dehydrogenase (*Bacillus cereus* ATCC 14579)
gi | 30022246	Leucine dehydrogenase (*Bacillus cereus *ATCC 14579)
gi | 30019663	nucleoside diphosphate kinase (*Bacillus cereus *ATCC 14579)
gi | 30021685	Oligopeptide-binding protein oppA (*Bacillus cereus* ATCC 14579)
gi | 30019304	ornithine-oxo-acid transaminase (*Bacillus cereus* ATCC 14579)
gi | 30022061	Pyruvate dehydrogenase E1 component alpha subunit (*Bacillus cereus* ATCC 14579)
gi | 168144706	response regulator aspartate phosphatase (*Bacillus cereus* G9842)
gi | 157690926	ribosomal protein S11 (*Bacillus pumilus* SAFR-032)
gi | 30018384	SSU ribosomal protein S19P (*Bacillus cereus* ATCC 14579)
gi | 42779595	tellurium resistance protein (*Bacillus cereus* ATCC 10987)
gi | 30022339	Transcriptional regulator, ArsR family (*Bacillus cereus* ATCC 14579)
gi | 30022700	Universal stress protein family (*Bacillus cereus* ATCC 14579)

**Table 2:** Proteins identified by MALDI-TOF/TOF MS.

Spot no.	Protein description	Accession no.	Score	Sequence coverage (%)	Peptide	Ion score
(A) *Bioactive factors *						
1	Cry1A(c)3	gi | 142742	342	25	IFTAFSLYDAR	94
3	proteinase VCA0223 (*Bacillus cereus *G9241)	gi | 47568584	166	21	R.NYAGSDTALQYAR.G	54
6	immune inhibitor A (*Bacillus thuringiensis*)	gi | 9858110	263	19	K.FEVVGQADDNSAGAVR.L	85
7	Immune inhibitor A precursor	gi | 124464	70	4	K.TYINQQIPDAGR.I	58
14	camelysin (*Bacillus thuringiensis *serovar *konkukian* str. 97-27)	gi | 49481531	84	21	K.EFLLQNSGSLTIK.D	56
40	Bacillolysin (*Bacillus cereus* ATCC 14579)	gi | 30023075	208	24	K.AAYLVSEGGDHYGVK.V	79
41	Cell envelope-bound metalloprotease (camelysin) (*Bacillus cereus *ATCC 14579)	gi | 30019432	102	30	K.DIFAPEWGEK.G	62
46	insecticidal crystal protein BTRX28 (*Bacillus thuringiensis *serovar *kunthalaRX28*)	gi | 13173240	104	15	K.NGIVLFHDIEVR.S	72
54, 53	camelysin (*Bacillus cereus*)	gi | 24474855	130	19	K.EFLLQNSGSLTIK.D	81
*	insecticidal crystal protein Cry2Aa (*Bacillus thuringiensis* serovar kurstaki)	gi | 47678765	131	22	R.GNSNYFPDYFIR.N	19

(B) *Metabolism *						
2	succinate dehydrogenase flavoprotein subunit (*Bacillus thuringiensis* str. Al Hakam)	gi | 118479669	245	32		
5, 20	transketolase (*Bacillus thuringiensis* serovar *konkukian* str. 97-27)	gi | 49478325	215	22	R.LVVLYDSNDISLDGDLNR.S	93
17	methylmalonic acid semialdehyde dehydrogenase (*Bacillus anthracis* str. Ames)	gi | 30262359	146	27	K.TVQGVIGSAFASSGER.C	59
18	adenylosuccinate synthetase (*Bacillus cereus* G9241)	gi | 47568666	88	11	R.VGDGPFPTELHDEIGHQIR.E	27
19	UDP-N-acetyl-D-mannosamine 6-dehydrogenase (*Bacillus thuringiensis* serovar* israelensis* ATCC 35646)	gi | 75760242	128	8	K.VGEDFYIGYSPER.I	54
21, 22	isocitrate dehydrogenase (*Bacillus cereus* ATCC 14579)	gi | 30022668	309	27	K.DSIADIFLQQILTRPR.E	80
36	thiamin biosynthesis ThiG (*Bacillus cereus* G9241)	gi | 47564664	226	27	K.AIDVSEAEILTFAVR.R	88
42	fructose-bisphosphate aldolase (*Bacillus cereus* G9241)	gi | 47568789	298	25	K.LDSIEDALNITYNALQEGAIGVDMGR.N	161
52	3-ketoacyl-(acyl-carrier-protein) reductase (*Bacillus cereus* ATCC 14579)	gi | 30020097	86	19	K.FGVLGLTESLAMEVR.K	55
34	malate dehydrogenase (*Bacillus anthracis* str. Ames)	gi | 30264663	145	12	R.YSYAGGIPLETLIPK.E	72

(C) *Crystal and spores formation *						
12	oligopeptide ABC transporter, substrate-binding protein (*Bacillus thuringiensis* serovar *konkukian* str. 97-27)	gi | 49481243	101	13	K.GIANHTFGGDFSYK.W	50
13	Oligopeptide-binding protein oppA (*Bacillus cereus* ATCC 14579)	gi | 30021687	195	30	K.TLLEEDVAIVPLYQR.G	70
24, 33	stage IV sporulation protein A (*Bacillus cereus* G9241)	gi | 47565971	226	21	K.IEYDQVADALR.M	77
30	Threonine synthase	gi | 135811	307	40	K.GHVIEEPETIATAIR.I	94
43, 47	CotJC protein (*Bacillus thuringiensis *serovar *israelensis *ATCC 35646)	gi | 75760539	373	52	K.LLIEQYGGADGELAAALR.Y	92
56, 57	Uncharacterized 20 kDa protein in cryB1 5′ region (ORF1)	gi | 140467	143	26	K.YSVQQLPHYVIDGDHIQVR.E	

(D)* Amino acid and protein synthesis *						
4	5-methyltetrahydropteroyltriglutamate-homocysteine methyltransferase (*Bacillus cereus* ATCC 14579)	gi | 30022091	298	29	R.YQEEIGLDVLVHGEFER.T	102
10	bifunctional GMP synthase/glutamine amidotransferase protein (*Bacillus anthraci*s str. Ames)	gi | 30260444	93	26		
11	1-pyrroline-5-carboxylate dehydrogenase (*Bacillus anthracis* str. Ames)	gi | 30260481	99	14	K.AREDFHVGNLYFNR.G	33
26	elongation factor Ts (*Bacillus cereus *ATCC 14579)	gi | 30021914	184	52	K.TDADAFGAYLHMGGR.I + Oxidation (M)	22
27	translation elongation factor Ts (*Bacillus cereus* G9241)	gi | 47569104	78	5	K.TDADAFGAYLHMGGR.I	73
35	Cysteine synthase (*Bacillus cereus *ATCC 14579)	gi | 30018339	92	21	K.EHGYFIPQQFK.N	38
37	branched-chain amino acid aminotransferase (*Bacillus anthracis* str. Ames)	gi | 30261496			K.SLNYLNNILVR.I	56
45	transcriptional repressor CodY (*Bacillus cereus* ATCC 14579)	gi | 30021916	216	40	R.ELFGQGLTTIVPIVGGGER.L	76
51	30S ribosomal protein S4 (*Bacillus cereus* ATCC 14579)	gi | 30022730	167	39	R.SELPAEINEALIVEFYS.R	44

(E) *Other functions *						
8, 9	5′-nucleotidase (*Bacillus thuringiensis* serovar israelensis ATCC 35646)	gi | 75761007	395	47	K.GANFPYVAANFYNK.S	85
15	ATP synthase subunit B (*Bacillus cereus* ATCC 14579)	gi | 30023337	257	64	R.ALSPEIVGEEHYEVAR.Q	75
16	Peptidase T (*Bacillus cereus* ATCC 14579)	gi | 30022227	232	34	R.FEGGTQTNIVCDHVQIFAEAR.S	102
23	electron transfer flavoprotein, beta subunit (beta-ETF) (*Bacillus thuringiensis* serovar *konkukian*)	gi | 49481481	527	66	R.DAQGGEVTVVTVGGEDSEKELR.T	148
25	NAD(+) synthetase (*Bacillus anthracis* str. Ames)	gi | 30262026	254	38	K.GFVLGISGGQDSTLAGR.L	104
28	aminomethyltransferase (*Bacillus cereus* ATCC 14579)	gi | 30022309	326	41	K.YAAVDTEVEIEIR.N	76
29	acyl-CoA dehydrogenase (*Bacillus cereus *ATCC 10987)	gi | 42781609	213	61	K.TAEFPYETFQK.M	51
31	ketol-acid reductoisomerase (*Bacillus anthracis* str. Ames)	gi | 30261892	413	58	R.HSISDTAEFGDYVTGSR.I	86
32	3-ketoacyl-CoA thiolase (*Bacillus thuringiensis* serovar *israelensis* ATCC 35646)	gi | 75760332	156	17	R.YCSSGLQSIAYGAER.I	64
38	organic solvent tolerance protein (*Shewanella oneidensis* MR-1)	gi | 24375135			K.INSEEEWGEIWNAK.L	56
39	inosine hydrolase (*Bacillus thuringiensis* serovar *kurstaki*)	gi | 156081515	217	23	K.LIEPCPEDIIIVATGR.L	80
44	purine nucleoside phosphorylase (*Bacillus cereus* ATCC 14579)	gi | 30019611	144	30	K.YIAETFLEDVTCYNNVR.G	80
48	hypothetical protein BC1708 (*Bacillus cereus *ATCC 14579)	gi | 30019852	225	45	K.NGIVLFHDIEVR.S	65
49	Phage shock protein A (*Bacillus thuringiensis* serovar* israelensis* ATCC 35646)	gi | 75758863	286	57	K.ILFEEQEALVK.K	42
50	hypothetical protein BC2244 (*Bacillus cereus* ATCC 14579)	gi | 30020376	116	25	K.YGQEVTQYEQLAR.Y	88
55	Alkyl hydroperoxide reductase C22 (*Bacillus cereus* ATCC 14579)	gi | 30018585	123	37	R.TITTNFNVLMEEEGLAAR.G	70

*The protein was separated by SDS-PAGE.
